# Toward Alleviating the Stigma of Hearing Aids: A Review

**DOI:** 10.3390/audiolres14060087

**Published:** 2024-12-04

**Authors:** Edward Madara, Achintya K. Bhowmik

**Affiliations:** 1 St. Paul’s School, Concord, NH 03301, USA; edward.madara@sps.edu; 2 Starkey Hearing, Eden Prairie, MN 55344, USA; 3 Department of Otolaryngology-Head and Neck Surgery, Stanford University School of Medicine, Stanford, CA 94305, USA; 4 Wu Tsai Neurosciences Institute, Stanford University, Stanford, CA 94305, USA

**Keywords:** hearing aid, hearing loss, adoption rate, stigma, design, functionality

## Abstract

Despite the significant advancements in hearing aid technology, their adoption rates remain low, with stigma continuing to be a major barrier for many. This review aims to assess the origins and current state of hearing aid stigma, as well as explore potential strategies for alleviating it. This review examines the societal perceptions, psychological impacts, and recent technological advancements that can influence hearing aid adoption and reduce stigma. **Methods:** A narrative-focused review of the literature from peer-reviewed journals and reputable sources was conducted, analyzing papers on hearing aid stigma, adoption rates, and technological solutions. The research works were categorized based on their focus on the drivers and alleviation strategies for the stigma of hearing aids. **Results:** This review identifies stigma as a complex, multifaceted issue driven primarily by ageism, disability perception, and the association of hearing aids with aging and incapability. Despite technological improvements, the studies surveyed listed stigma as a major factor in non-adoption. Technological advancements such as artificial intelligence in sound processing, multifunctional features, and innovative design have shown potential in reducing stigma and improving user experience. **Conclusions:** Alleviating the stigma of hearing aids requires a multi-pronged approach, combining improvements in technology with changes in societal perceptions. Multifunctional devices including both health and communications functions, advanced signal processing, and esthetic improvements can drive their adoption, but broader public health awareness and education are also essential to changing societal attitudes and fostering greater acceptance of hearing aids.

## 1. Introduction

The human sense of hearing is an extraordinary capability, allowing us to perceive and interpret a vast array of sounds from the environment, from the subtle rustling of leaves to the nuanced tones of speech and music. Our ears are sophisticated biological sensor systems that transduce sound waves into neural impulses. The ears and brain work in tandem to process these sounds effortlessly, enabling us to locate their sources, interpret complex auditory signals, and even filter out background noise, all without conscious effort [1]. This remarkable sense operates continuously, often unnoticed, as we navigate through our daily lives, and is often taken for granted as long as it functions normally. It is only when our hearing is impaired that we begin to appreciate its vital role in our connection to the world around us.

Hearing loss may be the world’s largest health crisis that many fail to notice, care about, or mention. According to the World Health Organization, a staggering 1.5 billion people globally suffer from some type of hearing loss (≥20 dB elevated hearing thresholds at audible sound frequencies) [2]. Of this hearing-impaired population, nearly half a billion live with “disabling” hearing loss, meaning that to live a normal life, they require hearing assistance.

Hearing loss, if left untreated, also has significant knock-on effects or comorbidities. According to Shukla et al., who conducted a comprehensive survey regarding the mental health impacts of hearing loss, untreated hearing loss forces those afflicted into social withdrawal, as they are therefore unable to participate in normal and routine conversations [3]. That social withdrawal coupled with the other cerebral detriments of hearing loss, such as dementia, could be one of the most significant causes of cognitive decline in elderly people [2,3]. Outside of mental health, hearing loss also has a proven comorbidity with obesity and cardiovascular diseases, as hearing-impaired persons are more likely to not participate in social events, and therefore become highly sedentary [4]. Patients with hearing impairment have been shown to suffer from significantly increased risks of falls [5]. Not only does hearing loss drastically undermine the health of hearing-impaired individuals, but because of its detriments to cognition and intelligence, one trillion “international dollars”, are lost because of it yearly, according to the World Health Organization [2]. Hearing loss, clearly, from a public health and economic perspective, could be one of the world’s most perilous conditions.

Sensorineural hearing loss (SNHL) is the most common form of hearing impairment, accounting for nearly 90% of all hearing loss cases by some estimates [6]. The other 10% is conductive hearing loss (CHL) of which earwax buildup is the most common subset, in addition to pathologies of the tympanic membrane and the middle ear structures [7]. Sensorineural hearing loss largely relates to either the damage or de-innervation of cilia hair cells which transform sounds to become understandable electrical signals that are relayed to the auditory cortex of the brain in the form of nerve impulses. Simply put, SNHL is derived from issues with the inner ear. Treatments for SNHL include both hearing aids and cochlear implants, but some estimates account for 80% of SNHL cases being treatable with hearing aids [6]. These patients have hearing loss that ranges from mild up to severe, accounting for more than 1 billion people [8]. The other 20% of the SNHL patients could be cochlear implant candidates. Cochlear implants are surgically placed medical devices that electronically stimulate the auditory nerve, acting as an artificial cochlea. Cochlear implants are a great solution for those who are severely or profoundly deaf yet, because of their narrow patient focus and significantly higher costs due to surgical procedures, they are not as ubiquitous as hearing aids [6].

Unfortunately, many people delay seeking help until their hearing impairment severely impacts their daily life, despite the availability of hearing aids that can enhance communication, social interaction, and overall well-being. This gap underscores the need for increased education, accessibility, and support to encourage more individuals to take advantage of the hearing solutions available to them. According to the National Institute on Deafness and Other Communication Disorders, a surprisingly small percentage of Americans who could benefit from hearing aids actually use them, with estimates ranging from just 16% to 30% [9]. This disparity highlights a significant public health issue, as millions of individuals are missing out on the improved quality of life that hearing aids can provide. The reasons for this low adoption rate are multifaceted, including social stigma, cost barriers, a lack of awareness, and a general reluctance to acknowledge hearing loss as a problem. In this paper, we review the stigma associated with hearing aids and examine the ways to alleviate it with improvements in design and increased functionality.

## 2. Methods

To conduct the research for this paper, a narrative review approach was adopted. This method was chosen to allow for a comprehensive exploration of the topic, encompassing a wide range of research sources beyond a narrow selection of journals or databases. The goal was not only to analyze an extensive body of work but also to draw meaningful inferences to propose potential solutions for the stigma surrounding hearing aids. The narrative review approach offered the flexibility to include valuable insights from respected sources beyond peer-reviewed articles, enriching the analysis with diverse perspectives.

The review process involved systematic online searches aimed at identifying and organizing research related to the stigma influencing hearing aid adoption and acceptance, and methods to alleviate the stigma. PubMed, provided by the National Institutes of Health, served as an important database that was utilized in this research due to its extensive coverage. Additionally, numerous other journals and publications were examined for their relevance and contribution to the topic. Articles were selected based on their quality, the rigor of their research methodologies, and their alignment with the paper’s objective of identifying the causes of hearing aid stigma and addressing them through improved designs and functionality.

The research was categorized into key themes, including the causes of stigma, sociological factors that either reinforce or mitigate stigma, and technological advancements aimed at increasing adoption and reducing stigma. The most significant peer-reviewed studies were tabulated, with their pertinent details and takeaways provided in the subsequent sections.

We acknowledge the potential for implicit bias in this paper, as well as the limitations of not conducting an exhaustive systematic review. However, we believe that the narrative review framework is the most suitable approach for synthesizing and presenting this research in a way that aligns with our objective of addressing hearing aid stigma. This method allows for a nuanced and holistic examination of the topic, emphasizing both depth and breadth in the analysis.

## 3. Survey of Prior Research

### 3.1. Justification of the Review

In the currently existing literature, there are several review papers examining the stigma of hearing loss, hearing aid adoption, and their use [10,11,12]. Our paper, however, differentiates itself from the other publications because of its composition as a narrative review. It presents itself more easily, following a framework that the general audience could follow and understand, and it also provides a comprehensive overview of strategies toward solving the issue of hearing aid stigma, from the root cause to the possible alleviators. To that end, we have structured the presentation as comprehensive, thematic, and advisory.

Among the existing review articles, Ekberg and Hickson’s paper, “To tell or not to tell? Exploring the social process of stigma for adults with hearing loss and their families: introduction to the special issue”, provides systematic and scientifically sound evidence for hearing aid stigma, but it simply focuses on its presence, providing few further implications to their study [10].

Ruusuvuori et al.’s paper, “Studies on stigma regarding hearing impairment and hearing aid use among adults of working age: a scoping review”, is closer to what we are attempting to accomplish. However, the systematic nature of their article leads them to simply prove that hearing aid stigma can be evaluated as a social process, without explaining some of its other root causes that are crucial to understand. In addition, even though their study provides some of the same further implications as ours, it simply states them in the conclusion, instead of giving broad, in-depth explorations and explanations of them as their discussion [11].

Ng and Loke’s paper, “Determinants of hearing-aid adoption and use among the elderly: A systematic review”, accomplishes one of the sub-objectives of our paper, discovering the root causes of hearing aid stigma; in fact, their paper could have replaced a long list of other references that accomplish something similar. However, that is their only objective. Unlike our paper, they do not provide potential solutions to hearing aid stigma, in addition to their systematic research on the underlying causes [12].

### 3.2. Narrative Review of Literature

The body of research on hearing aid stigma offers a comprehensive understanding of the various factors contributing to the non-adoption or underuse of hearing aids, despite their potential to significantly enhance quality of life. In this section, we will review key studies that have analyzed the social, psychological, and technological dimensions of hearing aid stigma. Table 1 provides a summary of notable research works, organized by authors, publication details, and their focus areas. These studies provide insights into both the causes of stigma—such as ageism, perceptions of disability, and concerns about esthetics—and explore potential solutions, including advancements in technology and design. This survey aims to contextualize the current state of hearing aid adoption and identify pathways to reducing the associated stigma.

### 3.3. Is There a Stigma?

In short, the surveys reveal that there continues to be a stigma associated with hearing loss as a whole, but especially regarding hearing aids as the main solution to hearing loss. A multitude of papers, spanning two decades, and multiple continents, state stigma as either the primary cause of hearing aid non-adoption or one of the leading causes.

Most recently, Alcido, writing for Forbes Health, conducted a survey on the limited adoption of hearing aids [13]. She concluded that 46% of hearing-impaired people do not wear a hearing aid. More pertinently, however, out of the entire survey pool, 48% of people believed that hearing aids are stigmatized. The stigma, Alcido noted, was rooted in a fear of aging and incapability (i.e., people do not want to be perceived, nor do they want to perceive themselves as old or disabled). Importantly, and optimistically, however, the Forbes survey also demonstrated that hearing aid stigma was less prevalent in the younger generations, indicating that the stigma could naturally decrease over time.

As somewhat of a foil to Alcido’s survey, McCormack and Fortnum’s 2013 study for the International Journal of Audiology as well as Alvarado’s 2011 article for Audiology Research were also reviewed [14,15]. Those papers offered international and historical insights into hearing aid stigma to examine its evolution and global expanse. These two research works were conducted through rote mathematical anthropological analysis. McCormack and Fortnum observed the current hearing aid users in the United Kingdom. Though they found the quality to be the greatest inhibitor to regular use for the users who already owned hearing aids, they found that stigma was the largest barrier to purchasing hearing aids in the first place. Alvarado compared hearing aid users in the United States and Italy. Though they found the cost to be the largest barrier, cosmetic and social reasons for hearing aid non-adoption (all of the individual factors that make up stigma), occupied more of the highest-ranked positions overall. Additionally, cost was only a factor in the United States, as Italy has a large public healthcare system that subsidizes hearing aids.

In a larger and more quantitative approach to hearing aid analysis, Kochkin’s examination of the MarkeTrak VII survey on hearing loss also saw stigma as a significant inhibitor [16]. Kochkin concluded that “nearly half (48%) [of the potential hearing aid patients surveyed] indicated that stigma contributed to their desire not to wear hearing aids”. Apart from the cost and a perceived lack of need, which could also be attributed to stigma, stigma alone was the largest factor relating to hearing aid non-adoption. Additionally, though her paper is more poignant when examining what causes the stigma of hearing aids, one quotation from Wallhagen’s 2009 paper discussing the stigma of hearing loss as a whole is highly important in evaluating the presence of stigma [17]. She found that “even if hearing aids were given away by the government at no cost to the user, 65% of the hearing-impaired population would decline the offer”.

### 3.4. What Causes the Stigma?

As a distinctly psychological and sociological issue, there are many factors and facets of hearing aid stigma, yet all of these factors can be roughly categorized as systemic and societal ageism and ableism.

**Figure 1:**
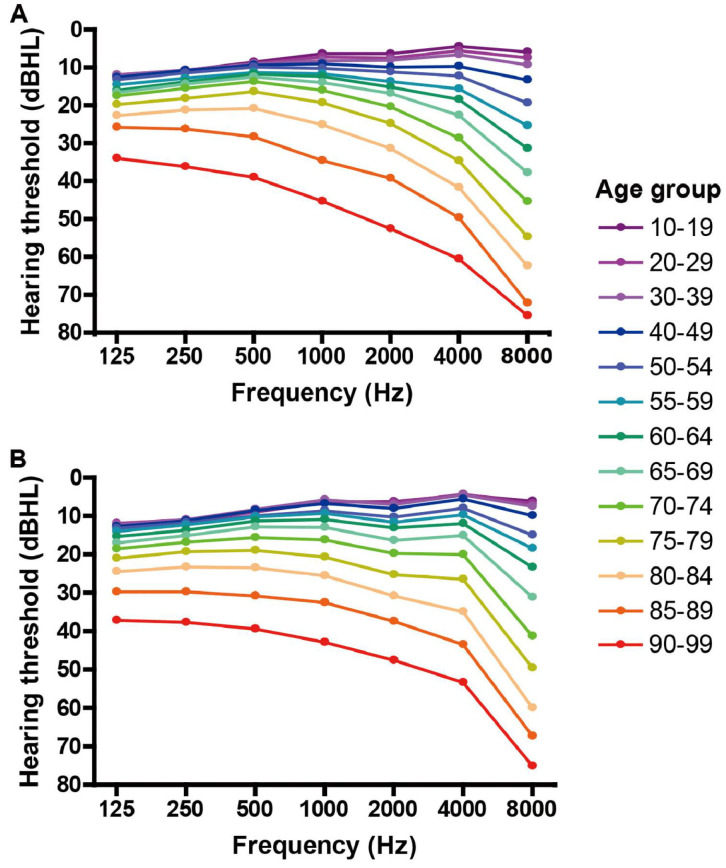
The age-related progression of hearing loss across different frequencies for men (**A**) and women (**B**). The data, collected from over 10,000 individuals aged 10 to 99 years in Japan, show that hearing thresholds progressively increase (indicating worse hearing) with age, particularly at higher frequencies [20]. Re-used with permission.

Though Sindi et al.’s paper for Cureus Journal does provide a wide overview of hearing aid stigma, it provides an analysis of the stigma, differentiating it from the articles above [18]. Sindi et al. presented their participants (*n* = 517) with various pictures of hearing people, and hearing-impaired people, who were hearing aid users, and asked them if they believed that the hearing-impaired people seemed disabled or “required additional assistance”. They found that 40.3% of people (*n* = ~208) believed that hearing aid users fit the aforementioned description. Their survey thus points toward ageism as a root cause of the stigma. Numerous research studies have established that the prevalence of hearing loss indeed progresses with age, and it is a global phenomenon. A study on the prevalence of hearing impairment in the United States in 2019 shows that approximately 30% of people in the age group of 50 to 59 years suffer from hearing loss, which increases to over 70% for the age group of 70 to 79 years, and exceeds 85% for people over 80 years [19]. As another example, Figure 1 shows the summary of a retrospective study of over 10,000 Japanese men and women, between 10 and 99 years of age, and their hearing loss [20]. This study also shows the steady progression of hearing loss with age, with worse hearing thresholds at higher sound frequencies in older ages. On an optimistic note, just like the Forbes Health survey, Sindi et al. also claim diminishing stigma amongst younger generations. They attribute this transition to the prevalence of earbuds and the future designs of hearing aids.

Wallhagen’s 2009 paper entitled “The stigma of hearing loss”, is one of the seminal articles relating to its namesake subject, though it is somewhat dated [17]. Wallhagen attributes the stigma simply to the label of being hearing-impaired, as well as the convention of hiding a hearing impairment, in pursuit of a solution. She claims that hearing aid professionals who constantly push hearing aid discretion and invisibility could actually be empowering the stigma of hearing aids instead of their patients.

In a 2016 review article not dissimilar to this paper, David and Werner examined all of the surveys surrounding hearing aid adoption [21]. They found that 33.3% of all the surveys listed hearing aid stigma as the primary reason for non-adoption. Just like Wallhagen, they found that ageism and the pursuit of concealment were the main causes behind stigma.

### 3.5. How Can the Stigma Be Alleviated?

Though this point will be further elaborated in the analysis and discussion portions of this paper, hearing aid stigma can be categorized as observed or perceived. Observed stigma is impacted by design, and perceived stigma, which could be categorized as the “crowded room effect”, is affected by functionality.

Pullin’s book, *Design Meets Disability*, is an important resource to consider when assessing design and its impact on the observed stigma of hearing aids [22]. In this book, Pullin explores the impact of industrial designs and color palettes from popular consumer electronic devices, and how that might affect the stigma of hearing aids. Pullin also provides an anecdote of a teenager wearing a bright white, ostensibly visible hearing device, and how confident he seemed comparatively.

Kent, in a 2021 Medical Device Network article, also explores the potential for the intersection of design and hearing loss, however differently from Pullin [23]. Kent advocates for the individualism and customization of hearing aids, similar to glasses. She claims that it is far more important, for the sake of eliminating hearing aid stigma, to make hearing aids look good than to make them invisible, especially if invisibility trumps functionality. She also says that younger generations are especially attuned to this kind of design independence from the medical world.

Bhowmik and Fabry, in an IEEE Computer article, outlined the path forward for hearing aids to become multifunctional devices, transcending their primary purpose of amplifying sound [24]. The computational processing power in modern hearing aids has become so advanced, and the connectivity to the cloud so pervasive, that they are able to integrate fall detection and fitness tracking sensors, language translation capabilities, and virtual assistants into the hearing aids, in addition to audio streaming from devices such as smartphones, computers, and televisions. The impact of multifunctionality is that hearing aids can become devices that people want to wear instead of having to wear.

Artificial intelligence has significant capabilities within the audio world. In two papers, one scientific and the other clinical, Diehl et al., writing for Scientific Reports, and Jaekel and Xu in a Starkey Hearing white paper, assess the potential of deep neural networks (DNNs) based denoising algorithms as a major improvement in hearing aid performance and functionality [25,26]. They found that incorporating DNN algorithms reliably improved speech intelligibility scores, by increasing tbe signal-to-noise ratios (SNRs). In short, DNNs are a significant potential solution to hearing loss affecting the ability to understand speech in noisy environments. Just like in the Scientific Reports trial, Starkey’s work found success in speech intelligibility improvement when the DNN algorithms were enabled.

## 4. Analysis and Discussion

For as long as hearing aids have existed, users have struggled with the stigma of wearing them and the belief that wearing hearing aids indicates old age, frailty, and or disability [15,18]. This stigma can be observed (i.e., how a user thinks about themselves and the way their hearing aids might look) or perceived (i.e., how a non-user views the user or how a user thinks a non-user views them) [13,17,18]. Importantly, observed stigma often feeds perceived stigma. For example, in a Cureus Journal survey completed in 2023, 40% of the participants observed hearing aid wearers to either be “handicapped”, or “requiring extra assistance” to complete tasks. In order to avoid being viewed in such a way, many patients simply refused to wear hearing aids. This perceived stigma can be referred to as the “crowded room effect”—the desire by users when they walk into a crowded room to “fit in”, to not be the “odd person out”, or to not be “different”. A fear of aging and a desire to maintain a normal appearance are among the most significant reasons for stigma. The potent design and functionality of hearing aids can significantly abase both their observed stigma and their perceived stigma by fighting against the stereotypical correlation between aging and hearing aids.

Another study by Gallagher and Woodside identified the common themes influencing hearing aid adoption and use among older adults, including the attitudes toward hearing loss and the inadequacy of audiology services [27]. These findings suggest that providing better information and scheduling regular follow-up appointments could improve hearing aid adoption and use, with further research needed to optimize these interventions.

### 4.1. Assessment of State-of-the-Art Peer Research

Recently, the *Lancet* Commission on Hearing Loss (LCHL) began a process to evaluate the stigma of hearing loss, examining the facets of hearing aid stigma both quantitatively and qualitatively. This research has resulted in an excellent multi-paper special feature in *Ear and Hearing* [28]. In many ways, the *Lancet* Commission has similar research objectives to us, and our research has resulted in similar conclusions. We believe that our article stands as a shorter, qualitative, more succinct peer to the LCHL’s work.

The following is a summary of their introductory work as it pertains to our paper. The Commission first chose to define hearing aid stigma, in a variety of ways. They did so by surveying different groups of people who were either deaf or hearing-impaired, or people who were directly associated with those who were deaf or hearing-impaired (i.e., parents of a profoundly hearing-impaired child). They termed six different types of stigmata that could be felt: experienced stigma, perceived stigma, internalized stigma, anticipated stigma, observed stigma, and secondary stigma. As many people experience more than one of these types, the commission also coined “intersectional stigma”, to describe any combination of the aforementioned stigmata. The quantitatively derived definitions of stigma were important because they laid the groundwork for specific research into each of these stigmata, upon which specific solutions can be found. Our research, since it focuses on what the LCHL would describe as “perceived” or “observed” stigma, finds solutions to these two stigmata in the advanced design and functionality of hearing aids.

### 4.2. Observed Stigma and the Impact of Design

In its most basic form, a hearing aid consists of microphones, picking up sounds from the wearer’s environment and transforming them into electric signals; an amplifier, increasing the power of those signals and processing them; and a receiver, more commonly known as a speaker, playing those signals into the ear canal; and a battery powering the device [29]. Modern hearing aid designs have refined these basic components into highly sophisticated, miniaturized devices that offer a range of customizable features and styles to meet individual needs. Advances in digital signal processing allow for precise amplification and noise reduction, while modern receivers deliver clearer, more natural sound. Additionally, contemporary hearing aids often incorporate wireless connectivity, enabling seamless integration with smartphones and other devices for streaming audio [24].

**Figure 2:**
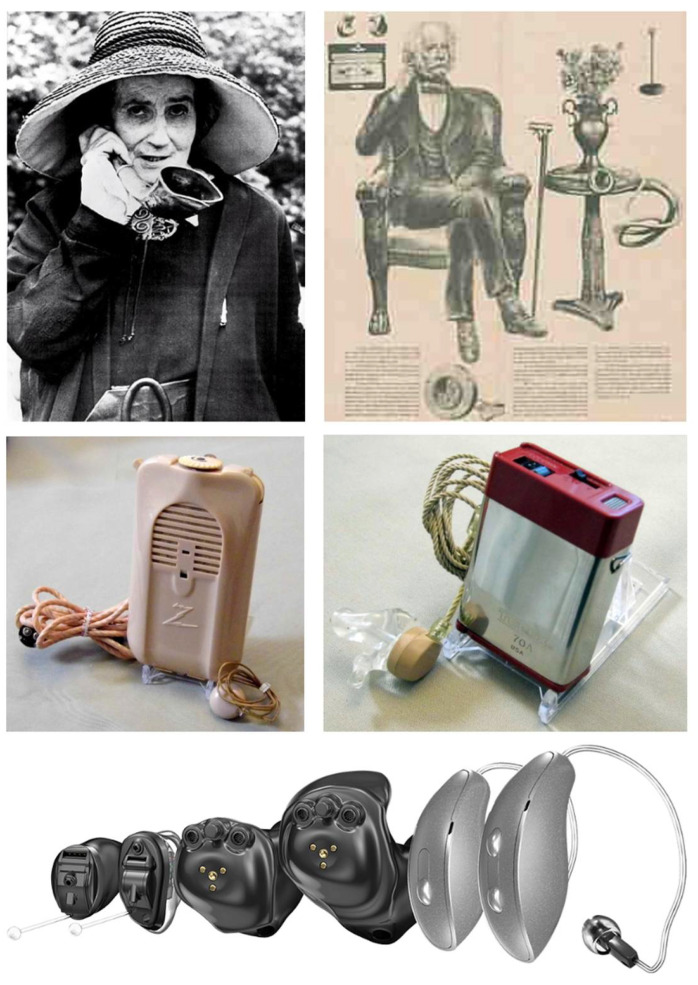
Evolution of hearing aid form factors. (**Top left**): Madame de Meuron with an ear trumpet, an early form of hearing aids dating back to the 17th century. (**Top right**): Frederick Rein’s acoustic chair, designed for King John VI of Portugal in the early 19th century. (**Middle left**): body-worn hearing aids based on the vacuum tube, circa 1944. Middle right: transistor body-worn hearing aids, circa 1979. (**Bottom**): modern hearing aids in 2024. From (**left**) to (**right**): invisible in-canal (IIC), completely in-canal (CIC), in-the-canal (ITC), in-the-ear (ITE), micro receiver-in-canal (Micro RIC), and receiver-in-canal (RIC) designs, all from Starkey Hearing. Re-used with permission.

As depicted in Figure 2, at their inception, hearing aids were rudimentary and mechanical systems, relying on passive amplification techniques. The ear trumpet of Madame de Meuron, for example, was a simple, funnel-shaped device that captured sound and directed it into the ear, while Frederick Rein’s acoustic chair for King John VI of Portugal incorporated hidden sound-collecting tubes into a piece of furniture, allowing the user to hear conversations more clearly without the need for a conspicuous device. The mid-20th century marked a major milestone with the advent of body-worn hearing aids utilizing vacuum tube technology, a significant leap in portable amplification devices around the 1940s. This was further refined by the 1970s with the introduction of transistor-based body-worn hearing aids, which offered greater miniaturization and efficiency.

Though the initial effects of these hearing aids seemed positive, an inability to distinguish signals (voices and other important sounds) from noise (everything else), made these hearing aids, and indeed many low-end digital hearing aids, largely inadequate. Today’s state-of-the-art hearing aids, however, are far more advanced than the rudimentary sum of their parts. Currently, hearing aids make use of digital signal processing techniques within the amplifier, making the sound passed on to the wearer far better and clearer. Also, at their earlier iterations, hearing aids were large, obtrusive, and were required to be worn on the body with wires connecting to the earpieces. Now, as all of the components that make up hearing aids have become far smaller, hearing aids can be made to be completely invisible inside the wearer’s ear canal in invisible-in-canal (IIC) or extended-wear designs, or nearly invisible form factors such as completely in-canal (CIC) and receiver-in-canal (RIC) styles, or more conspicuous designs such as in-the-canal (ITC), in-the-ear (ITE), or the traditional behind-the-ear (BTE) forms [24,29,30].

For reasons of simple necessity, the design of hearing aids has often reflected their functional use. The most ubiquitous design form currently is the RIC, where the speaker, referred to as the receiver, is placed in the ear canal and the device, consisting of the battery pack, microphones, and processors, sits behind the ear, connected by a thin translucent conducting wire over the top of the ear. This form has been effective in aggregating the component parts of hearing aids, but their visual appearance has typically still been perceived as medical in nature [29]. Contrast this to the emergence of consumer electronic earphones or earbuds, such as the Apple AirPods launched in December 2016. These devices, while not as medically advanced or effective as prescription hearing aids, are now worn by over 100 million people worldwide [31] and “carry none of the social stigma sometimes associated with hearing aids”. However, it must also be considered that these earphones are occluding devices with limited battery life, and as such cannot replace hearing aids for their comfortable all-day use. In addition, consumer electronic earbuds come with their own form of stigma. Will it be socially acceptable to wear them at a family dinner or in a business meeting?

The potential, however, exists for an improvement in the design form of hearing aids to improve the observed stigma. This opportunity also extends to the style of hearing aids. For years, the eyeglass industry fought against a similar visual stigma that was observed and perceived [32]. This stigma was popularized by the fact that Clark Kent takes off his glasses to become Superman [33]. Then, starting in the 1970s, eyeglass manufacturers turned to thermoset plastics and exotic materials to revolutionize the design style of glasses [22]. As of 2024, more than 4 billion people wear glasses [34]. Vision-impaired people have, in many cases, become proud of their glasses. As the glasses industry revolutionized, the lives of glasses wearers improved. The same opportunity exists for hearing device designers. If the hearing-impaired population can like wearing hearing aids, and not feel denigrated while wearing them, then they will wear their hearing aids more frequently, improving their quality of life and diminishing their risk of hearing loss comorbidities.

### 4.3. Perceived Stigma and the Impact of Functionality

Improving the observed stigma of hearing aids will increase the likelihood of adoption as self-acceptance promotes usage. But, how about the perceived stigma—the reluctance to walk into a crowded room wearing hearing aids for fear of being viewed differently? One answer lies in increasing the number of people in the room wearing them. The desire, or necessity, of wearing hearing aids is driven in large part by their functionality [13]. Hearing aid functionality has two core parts: sound quality and feature richness. Hearing aids with excellent sound quality should be able to capture, amplify, and deliver sound to the user nearly as though they had normal hearing. Sound quality also necessitates limiting the feedback within the ear canal and isolating speech in noisy scenarios. This quality is now based on advanced digital sound processing, some of which is powered by neural networks [24,25,26]. Hearing aids that are feature-rich are multifunctional. Capitalizing off of the unique biology of the ear canal, as well as the fact that a hearing aid sits in the ear, hearing aids now can have body tracking sensors, fall detection, and the ability to provide language translation and personal voice assistant functions, improving the wearing experience of the user [24]. In turn, more people should want to wear hearing aids, driving up their adoption rate. Thus, more people in a “crowded room” will be wearing hearing aids, lessening the stigma of standing out in a crowd of otherwise non-hearing aid wearers.

The design and functionality of hearing aids are not completely independent, either. The ergonomics of hearing aids are one of the elements that cross over between those two categories. Many older and hearing-impaired patients also struggle with dexterity issues or neuropathy. Though those patients might desire discreet, small, even invisible hearing aids, those hearing aids are impossible for them to use. Therefore, the larger, easy to manipulate, simply designed full shell ITE hearing aids, which, consequently, might show more, are also crucial [14]. In addition, improper placement, inadequate maintenance, and insufficient follow-up or counseling with hearing care professionals significantly impact hearing aid use, often exacerbating stigma and leading patients to cite stigma as a reason for non-compliance.

### 4.4. Toward Alleviating the Stigma

De-stigmatizing hearing aids, like many things, starts with the device designers and ends with the consumer. Having spent decades improving the quality of hearing, designers now have the opportunity to combine form with function and deliver products that users are excited to wear. Depending on the form factor used, hearing aids can be customized with the user’s favorite color or pattern, branded with their favorite luxury marque, or reflect their alma mater, or favorite artist. All of this holds the potential to lower the stigma of hearing aids by allowing the user, for perhaps the first time in their life, to “show off” and “be proud” of wearing them. In this way the observed stigma of hearing aids can begin to abate the same way the observed stigma of eyeglasses has greatly diminished over time. In his presentation to the Microsoft Research organization, Graham Pullin, while describing work by Charles and Ray Eames, said that “designers from art school backgrounds can enrich” design for disabilities, as that culture can make more interesting and, possibly, more functional designs that are as beautiful as they are useful [35]. He even said that “a teenager wearing a white hearing aid, which was more visible than a flesh-pink one, looked self-confident wearing it”. This encounter mirrors how hearing aid stigma can potentially be averted through accentuation and great design [22]. When that happens, the stigma for the use of these disability-aiding assistive products may be reduced. In turn, the effects of those disabilities are diminished, and the quality of life increases for those afflicted. Clinical problem-solving need not be absent from hearing aid design, as this ensures that form does not overtake function, but there should be some element of beauty and fashionable sophistication that is also present. Significant strides are being made in this direction. For example, state-of-the-art modern hearing aids are starting to offer more visually attractive and comfortable designs than the traditional devices of the past [24].

**Figure 3:**
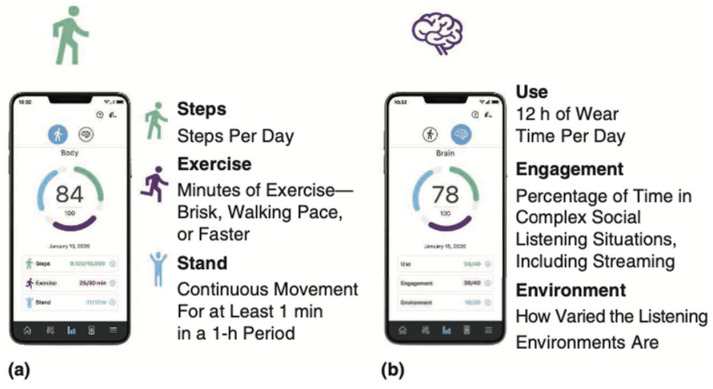

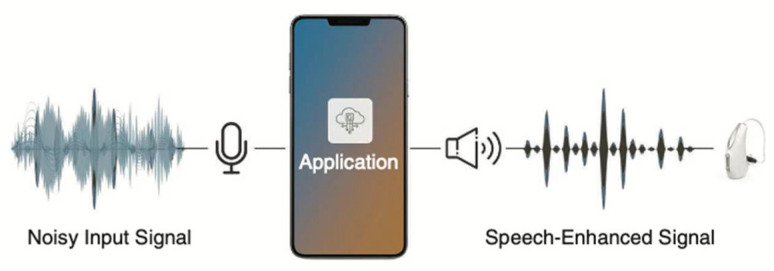
Demonstrations of new features to expand hearing aid functionality. The first image is how the fitness tracking data from Starkey’s hearing aids is shown to the wearer. The second image is a demonstration of how their first DNN-based denoising software worked [24]. The current generation of advanced hearing aids from Starkey incorporates a DNN accelerator embedded within the processor of the devices.

Functionality is equally important, as it eliminates the perceived stigma of hearing aids by making them devices that hearing-impaired people want to wear instead of having to wear. Advancements in features such as language translation, fall detection, geo-location, and fitness tracking have revolutionized the value of hearing aids to users and their families [24]. Today, there is no reason a user of advanced hearing aids should not feel as empowered, or more, in their life as an AirPods user does when they don their devices. Figure 3 shows the digital demonstrations of these functionalities, either through their fitness tracking software or their voice-enhancement algorithms. As hearing aids become more valuable to the user, their adoption rate should rise. This rise, in turn, should increase the number of people in a crowded room wearing hearing aids and the perceived stigma (if even still present) should fall.

In another example of a multifunctional assistive device, Deng et al. identified functional quality, perceived interaction speed, and ease of use as key factors enhancing communication effectiveness in their study of the use of augmented reality glasses as hearing aids [36]. This improved communication, in turn, fosters user confidence and a positive social image, which significantly influences the intention to adopt the glasses. These findings provide valuable insights for optimizing the design to enhance usability, social acceptance, and user confidence.

Dementia and cognitive decline due to a lack of interpersonal communication are two of hearing loss’s most significant comorbidities. Even for current hearing aid users, the difficulty in distinguishing speech from noise is one of their most common complaints, and this issue is perhaps the decisive factor influencing the aforementioned comorbidities. As such, many choose to go without their hearing aids, as they feel that they are not helping, furthering those problems. A program or device, therefore, that could effectively suppress noise, especially in loud environments such as restaurants, is critical. With modern advancements in artificial intelligence, including the development of algorithms based on the deep neural networks integrated in the hearing aid, rapid progress is being made in this direction [24,25,26,37,38]. Broadly, in a bibliometric analysis, Zhang et al. highlight the growing application of artificial intelligence in communication sciences and disorders [39]. While traditional machine learning methods dominate the field, there is an emerging shift toward deep learning approaches.

Voice isolation techniques are analogous to similar technologies used in the music industry. In that sector, producers have begun to use DNNs to isolate certain stems or instruments in a given track of music. By using time-to-frequency domain transform (TFDT), and then allowing a DNN to recognize the patterns in the frequency signatures of sounds, producers can isolate and tune a specific instrument. Likewise, though it is more difficult to isolate speech than music, this same process can be used to isolate voices from noise [40]. As an example, IRIS Audio applied voice isolation software to call centers and other noisy mission-critical environments where clear communication is critical [41]. An example of the effectiveness of DNN-based voice isolation is a University of Washington experiment, where they tuned regular, noise-canceling headphones to isolate voices and suppress all noise except for the sole person to whom the subject wanted to listen [42]. The advent of voice isolation technologies alone is not groundbreaking for solving the stigma of hearing aid; rather, it is simply a new function that could drastically improve the experiences of hearing aid wearers, allowing them to see the full benefits of hearing aids, solving their hearing loss.

Desai et al. explored user perspectives on improving hearing aids, identifying key areas for enhancement in physical design, sound quality, and service delivery. The participants expressed dissatisfaction with the hearing aid esthetics, fit, and functionality, emphasizing the need for greater user control and autonomy [43]. They also highlighted challenges with sound clarity in noisy environments and raised concerns about high costs and trust in hearing healthcare professionals. These findings suggest that future hearing aid designs should prioritize user-driven improvements, including better esthetics, enhanced sound clarity, affordability, and features that empower user self-efficacy and autonomy. If hearing aids were to become far better, functionally, then there is no doubt that more people would use them across the hearing loss spectrum.

Design and functionality alone can greatly reduce the stigma of hearing aids for both current hearing aid wearers or immediate candidates who are soon to make a decision on whether or not to begin using hearing aids. For the rest of the population, however, awareness needs to be raised about hearing aids, both in their current forms and in their possible future, more advanced forms. A study conducted by David et al. demonstrated that when non-hearing-impaired people watched videos about hearing aids, and the benefits that can be derived from them, they were both intrigued about hearing aids, either for themselves or for others and had a greater sense of sympathy for the hearing impaired and their use of hearing aids [21].

The results of that study clearly show the effectiveness of education against hearing aid stigma. As hearing aids become more advanced, therefore, public health organizations, as well as the hearing aid ecosystem could launch broad campaigns about the benefits of hearing aids, which should target influencing both the hearing impaired and the people surrounding them. Those campaigns could mirror other public health campaigns in their effectiveness and sympathy for those affected, similar to the Mothers Against Drunk Driving campaign of the 1980s [44]. Though those two campaigns are different in their focus, the elements of outreach and the design of the campaigns could be mirrored to ensure the success of a campaign for hearing aid adoption. The hearing aid awareness campaigns, if performed well, could be a major catalyst for the reduction in hearing aid stigma through design and functionality.

Finally, there are significant links between hearing loss and a poor quality of life, especially for older adults. As the World Health Organization stated in their World Report on Hearing, there are significant links between hearing loss and cognitive decline [2]. That quality of life, according to Dawes et al., manifests itself first as social isolation and depression, as hearing-impaired patients begin to withdraw from social interaction because they are unable to participate in conversation. Subsequently, that isolation leads to depression and significant loneliness [45]. All of these mental health problems (which can also lead to physical health problems, such as falling, derived from an inactive mind, according to Lin et al.), also according to Dawes et al., could be somewhat alleviated through hearing aid use [5,45]. In addition to all the benefits of hearing aid use, the consequences of the lack of hearing aid use must be addressed. Hearing loss, if left untreated, becomes a serious health problem, not just for the patient, but, if left to be promulgated, for society as well.

## 5. Conclusions

Hearing aids have undergone remarkable advancements in recent years, transforming from rudimentary sound amplification tools into sophisticated, multi-functional devices that utilize artificial intelligence to enhance sound quality and offer additional features like fall detection, physical activity, and social engagement tracking, and even language translation. Despite these innovations, the adoption rate of hearing aids remains strikingly low, with a very small fraction of people who need them actually using them.

In this paper, we have examined the pervasive stigma associated with hearing aids, which continues to be a significant barrier to their widespread adoption. We have explored how the stigma—both observed and perceived—can be alleviated by improving the design and functionality of hearing aids. By drawing parallels with the evolution of eyeglasses, which successfully transitioned from a stigmatized necessity to a fashion statement, this paper argues that hearing aids can similarly overcome their negative perception. By integrating multiple functions besides design improvements, hearing aids can transform into must-have health and communication devices that many would want to wear. This discussion also emphasizes the importance of public awareness and education in reducing stigma and increasing the acceptance of hearing aids, ultimately aiming to improve the quality of life for millions of individuals with hearing loss.

As hearing aid designers continue to create products that both look better and perform better, both the perceived stigma of wearing them and the observed stigma of being “disabled” will be reduced, paving the way to a world where hearing-impaired people are not discriminated against for wanting a better quality of life. As that happens, one of the world’s largest health crises, comprising one-fifth of the world’s total population, can be improved and those afflicted can receive the hearing care that they require and deserve.

## Figures and Tables

**Table 1:** Overview of reviewed papers, including authors, publishers, titles, publication dates, broad and specific focuses, and key findings relevant to the review.

Author, Publisher	Title, Date	Focus	Key Findings
Alcido, Macy; Forbes Health	Forbes health survey: Nearly half of people with hearing loss believe there is a hearing aid stigma; 16 April 2024	Is there a stigma? Assessment of American perception of hearing aids.	- 46% of hearing-impaired people do not wear a hearing aid. - 48% believe in a stigma with hearing aids. - The largest social barrier is hearing clearly in public spaces, i.e., airports (55% agreed). - A higher percentage of millennials (63%) believe there is a stigma, compared to Gen X (47%) and baby boomers (41%). - Stigma consists of fear of age/incapability. - Stigma prevalence equals non-adoption prevalence.
McCormack, Abby and Fortnum, Heather; International Journal of Audiology	Why do people fitted with hearing aids not wear them? 11 March 2013	Is there a stigma? Assessment of UK non-adoption of hearing aids (cost-subsidized environment).	- WHO statistics reveal that only 20% of people who would potentially benefit from wearing hearing aids actually do. - Hearing aid stigma/appearance prevents buyers from purchasing hearing aids, but does not prevent current owners from wearing them. - Quality of hearing aids, both in ergonomics and functionality, was the main stated non-adoption reason.
Alvarado, B.; Audiology Research	Perceptions about hearing aids from elderly non-users: a bicultural point of view (Italy and USA); 5 May 2011	Is there a stigma? Assessment of Italian and American perceptions on hearing aids.	- Cosmetic reasons for non-adoption of hearing aids were very prevalent, the second largest factor after cost. - Social factors about embarrassment and worry about other people’s perception were equally high, ranking second among the top five limiting factors. - Cost was a limiting factor in the United States, but not in Italy, as there is a significant subsidized public healthcare system.
Sindi, Abdullah; Hanbazazah, Kamal; Alamoudi, Malak M.; Al-Harbi, Ahd; Aljuhani, Mohammed; Zawawi, Faisal; Cureus	The hearing aid effect in the 2020s: Where do we stand? 19 April 2023	Is there a stigma? What causes the stigma? Analysis of current stigma.	- 40.3% of all survey participants (*n* = 517) determined hearing aid users to be disabled or “requiring extra assistance”. - Stigma is lessening over time, younger generations are more accepting of hearing aids. - Attributes the current state of stigma, and the future ability of stigma to lessen to prevalence of earbuds and the overall design of hearing aids.
Wallhagen, Margaret; The Gerontologist	The stigma of hearing loss; 10 July 2009	What causes the stigma? Analysis of current stigma.	- The label of being hearing impaired is the driving force behind stigma. People are scared of aging, of being disabled, they do not want to think of themselves as such, nor do they want others to. - Hearing professionals promising complete invisibility does not actually help stigma, it can further it by forcing people to hide it to receive quality treatment.
David, Danna and Werner, Perla; Stigma and Health	Stigma regarding hearing loss and hearing aids: A scoping review; 2016	What causes the stigma? Analysis and in-depth review of stigma.	- 33.3% of studies listed stigma as the primary reasons affecting the non-adoption of hearing aids. - The overarching stigma of hearing aids was ageism, the stigma against being elderly. - Concealing hearing impairment was the primary way that people fought against stigma, but that had other systematic communication issues.
Bhowmik, Achintya K. and Fabry, David A.; Computer	Hear, now, and in the future: Transforming hearing aids into multipurpose devices; 25 October 2021	What are solutions to hearing aid stigma? Evaluation of multifunctionality as a potential solution.	- Hearing aids, since their inception, have been single purpose devices. They have only served one purpose, with varying degrees of efficacy. - Modern signal processing has made hearing aids far more effective. Artificial intelligence (AI) has enabled hearing aids to provide a significantly clearer signal than before, greatly increasing speech intelligibility. - Given that hearing aids occupy the ear canal, they are poised to widen their scope toward being multifunctional devices, including language translation, voice assistants, fall detection, and fitness tracking. - When hearing aids are either so good that people want to use them, or they even become “cool”, adoption can rise with a diminishing stigma.
Diehl, Peter et al.; Scientific Reports	Restoring speech intelligibility for hearing aid users with deep learning; 15 February 2023	What are solutions to hearing aid stigma? Evaluation of deep neural network (DNN) based denoising as a potential solution.	- Speech understanding in noise has been one of the preeminent issues facing the hearing-impaired community, as well as hearing aid manufacturers. - State-of-the-art hearing aids have been integrating AI for nearly a decade, but noise-suppressing models have just come into existence. - DNN models were able to improve speech intelligibility significantly, increasing the signal-to-noise ratio (SNR) and suppressing background noise, even babble.
Jaekel, Brittany N. and Xu, Jingjing; Starkey	The many benefits of Edge Mode+: A multiplicity of measures reveal improved performance in hearing aid users; 2024	What are solutions to hearing aid stigma? How does a major hearing aid manufacturer’s efforts represent the advent of that solution?	- Starkey, one of the world’s preeminent hearing aid manufacturers, has been heavily investing in AI for their hearing aids over the past decade. - Edge Mode+ is Starkey’s attempt to deploy DNN based technology to enhance the signal-to-noise ratio for their hearing aids. - When Edge Mode+ was enabled, there was a significant increase in speech intelligibility scores.
Pullin, Graham	Design Meets Disability; 2009	What are solutions to hearing aid stigma? Evaluation of design and artistry as potential solutions.	- Hearing aid designs have traditionally prioritized their medical function over form and style. In contrast, eyeglasses have seen a massive reform in terms of designs, enhancing their stylistic appeals. - Pullin explores the possibilities of designing hearing aids to look good, and how that would affect wearers and the overall stigma of hearing aids.
Kent, Chloe; Medical Device Network	Hearing aid aesthetics: how the appearance of devices can impact adherence; 2 April 2021	What are solutions to hearing aid stigma? Evaluation of individualism and customization as well as artistry as potential solutions.	- Hearing aids, though they provide a significant increase in quality of life, are considered medical assistive devices. - Many hearing aid users want discreet hearing aids that make their hearing loss invisible, but that has downsides relating to the quality of the hearing aid’s functions and ergonomics. - Younger hearing aid users are taking a different approach to combating the stigma of hearing aids—accentuation. Instead of trying to hide their hearing aids, they are customizing them just like jewelry, with a similar level of individualism as glasses.
